# Dark Triad of Personality and Problematic Smartphone Use: A Preliminary Study on the Mediating Role of Fear of Missing Out

**DOI:** 10.3390/ijerph18168463

**Published:** 2021-08-10

**Authors:** Rocco Servidio, Mark D. Griffiths, Zsolt Demetrovics

**Affiliations:** 1 Department of Cultures, Education and Society, University of Calabria, Via Pietro Bucci, Building Cube 20/B, 87036 Arcavacata di Rende, Italy; rocco.servidio@unical.it; 2 International Gaming Research Unit, Psychology Department, Nottingham Trent University, Nottingham NG1 4FQ, UK; 3 Centre of Excellence in Responsible Gaming, University of Gibraltar, Gibraltar GX11 1AA, Gibraltar; zsolt.demetrovics@unigib.edu.gi; 4 Institute of Psychology, ELTE Eötvös Loránd University, 1064 Budapest, Hungary

**Keywords:** narcissism, psychopathy, Machiavellianism, fear of missing out, problematic smartphone use

## Abstract

The present study examined whether the relationship between the Dark Triad (DT) of personality and problematic smartphone use (PSU) can be explained by the mediating role of fear of missing out (FoMO). The role of FoMO in this relationship has yet to be examined. A total of 457 participants completed an online survey. Results indicated that males scored high on measures assessing DT of personality, while females scored high on PSU. Structural equation modelling showed that narcissism was directly associated with PSU. FoMO partially mediated the association between narcissism and PSU. Machiavellianism and narcissism were directly associated with FoMO. In the fully mediated model, narcissism (but not Machiavellianism) was still associated with FoMO, and in turn, FoMO was related to PSU. Although preliminary, the results of the present study indicated that Machiavellianism and narcissism might represent antecedents of FoMO, in addition to the Big Five personality traits, and both could be involved in the development of PSU.

## 1. Introduction

Despite the positive advantages of smartphones, their over-use can have detrimental effects on a minority of individuals (see [1], for a review). Problematic smartphone use (PSU) is a recent construct defined as excessive use of a smartphone with accompanying functional impairments in daily living, with symptoms resembling those found in substance use disorders [2]. Recently, it has been suggested that PSU shares some commonalities with the construct of internet-use disorder, since smartphones are the most common way for individuals to access online social media applications [3]. Empirical studies have indicated that PSU can lead to severe psychological impairments for a minority of individuals, including anxiety (see [4], for a review, low self-esteem [5], and low self-control [6,7]. Additionally, PSU has been associated with poor sleep quality [8] and poor academic outcomes [9].

However, smartphone use per se does not have necessarily negative effects on everyday life because individuals might become addicted to specific online channels and applications (e.g., social media) rather than the internet or smartphone itself [10]. Given the wide utilization of smartphones among the general population, there are still some concerning findings that have led researchers to investigate why some individuals are more disposed to develop a risk of addiction to these technologies than others. It is important to examine the antecedents of PSU because they can be incorporated into the design and implementation of more bespoke accurate prevention and intervention strategies.

According to the Interaction of Person-Affect-Cognition-Execution (I-PACE) model for addictive behaviors, individuals with different personality traits can develop different types and levels of internet-use disorders [11]. The I-PACE model has been applied to explore the role of Big Five personality traits on different kinds of problematic or internet-use disorders such as social media use disorder and PSU. Similarly, recent studies suggest that the Dark Triad (DT) of personality traits [12] is positively associated with problematic use of specific and nonspecific online activities [13,14].

Despite recommendations to explore the possible role of the DT of personality as predictors of fear of missing out (FoMO; see [15], for a review), to date, no previous study has yet explored this association. Consequently, the present study examined the effects of the DT of personality and FoMO on PSU. Overall, these constructs represent significant individual differences and the relationships among these variables with PSU have yet to be simultaneously investigated. Therefore, the present study explored the effects of the DT of personality on PSU and the mediating role of FoMO in this relationship.

### 1.1. Dark Triad of Personality and Problematic Smartphone Use

The DT of personality (i.e., [16]) is a collection of three interrelated, malevolent personality constructs: narcissism, psychopathy, and Machiavellianism [12]. More specifically, most studies have focused on the main validated dark traits [16,17] comprising narcissism (behaving ostentatiously, superiority, and grandiosity), psychopathy (antisocial and thrill-seeking behaviors, impulsivity, and lack of empathy), and Machiavellianism (manipulativeness and deceptiveness).

The DT of personality affects a variety of behaviors in different domains, usually in a way that is somewhat deviant. Prior research has examined the association between the DT of personality and the problematic use of technologies [18] and other online addictive disorders related to PSU [19]. For example, the results of a recent study suggested that the DT of personality may play a causal role in higher PSU (with different traits having diverse effects among gender), and that attachment styles partially explained the association between dark traits and PSU [13]. In a sample of university students, Kircaburun et al. [20] found that Machiavellianism and narcissism had small significant direct effects on problematic social media use, while the partial indirect effect of narcissism via self-esteem was significant, but the effect size was small. However, Demircioğlu and Göncü Köse [21] found no association between narcissism and Machiavellianism and social media addiction, demonstrating inconsistent findings with other previous studies. Moreover, research has also indicated that psychopathy is associated with impulsivity and emotion dysregulation, making it difficult for psychopaths to control their desires to spend time on their smartphones for pleasure and sensation-seeking purposes [13].

The results of another study indicated that social zapping (i.e., the inclination to cancel or switch between social schedules at the last minute in favor of more valuable alternatives) was mainly predicted by Machiavellianism and narcissism [22]. It should also be noted that the DT of personality traits are not maladaptive but are the result of an individual’s active adaptation to the environment [19]. Smartphones can be regarded as a technological tool that allows individuals to get access to modern services and applications. However, the relationship between the DT of personality traits and PSU needs to be further empirically explored as there is a paucity of data.

### 1.2. The Mediating Role of Fear of Missing Out

Fear of missing out is a psychological construct defined by an apprehension of being absent from other individuals’ rewarding experiences and the desire to stay connected with others’ experiences [23]. Previous studies have empirically investigated the reliability of FoMO as well as its relationship with social media addiction and PSU, among others (see [15], for a review). Additionally, other studies have reported direct and indirect associations of FoMO with PSU as well as its mediating role [5,24,25,26,27].

Seminal literature suggests that the Big Five personality traits [28] are antecedents of FoMO [25]. Drawing upon the I-PACE model [11], empirical evidence indicates that interrelated factors such as dispositional, environmental, and behavioral reinforcement systems interact with each other to develop internet use disorders and addiction to related technologies [14,20]. However, aside from the results that come from the aforementioned studies, the DT of personality traits has not been investigated in relation to FoMO.

Research exploring the extent to which the DT of personality traits predict social media use have largely focused on traits such as narcissism, typically indicating that individuals who score higher in narcissism are more frequent social media users (e.g., [29,30,31]) Since FoMO is related, among other online activities, to social media use [20], it becomes interesting to study its association with the DT of personality. Contrarily, Machiavellianism and psychopathy have shown inconsistent and contradictory results across studies [32], therefore further research in relation to these traits is required. Additionally, the DT of personality traits has distinct features that may generate vulnerability for problematic online behavior. Therefore, for the first time, the potential role of the DT of personality as determinants of FoMO was explored in the present study.

### 1.3. Hypotheses

There is preliminary evidence that the DT of personality predicts PSU [13]. However, no previous study has explored if the association between the DT of personality traits and PSU can be fully or partially explained by FoMO. Therefore, the aims of the present study were to (i) confirm the relationship between DT of personality (or single traits such as psychopathy and narcissism) and PSU; and (ii) examine the mediating role of FoMO in the relationship between DT of personality and PSU. Therefore, it was hypothesized that FoMO would mediate the association between the DT of personality and PSU.

## 2. Method

### 2.1. Participants, Procedures, and Ethics

A total of 462 Italian smartphone users was recruited online through a snowball sampling procedure. The initial sample comprised 121 males (26.2%) and 339 females (73.4%). The participants’ ages ranged from 18 to 38 years (*M* = 23.49, *SD* = 3.57). A preliminary screening of the data by computing the Mahalanobis distance with, *p* < 0.001 for the chi-squared (χ^2^) distribution value [33], resulted in the exclusion of five participants. Therefore, the final sample included 457 participants (120 males, 26.3%, and 335 females, 73.3%), aged 18 to 38 years (*M* = 23.49, *SD* = 3.57). Participants indicated they spent 3.15 h per day on social media platforms. Two participants did not report their gender and age.

All participants were informed of the study’s objectives and they were invited to complete an anonymous survey. Therefore, after indicating their consent for participation, participants began the online survey. All participants volunteered for the study and none of them received any kind of remuneration. Moreover, they were also allowed to withdraw their data from the study at any stage. This study was conducted according to the Helsinki Declaration and the ethical standards laid out by the Italian Psychological Association, in accord with the ethical regulations of the first author’s university ethics committee.

### 2.2. Measures

The Italian version of the Dark Triad Dirty Dozen scale [34] was used to assess Machiavellianism (e.g., “I tend to manipulate others to get my way”; α = 0.84), psychopathy (e.g., “I tend to lack remorse”; α = 0.63), and narcissism (e.g., “I tend to want others to admire me”; α = 0.81). Items (from 0 = “not at all” to 4 = “very much”) for each sub-scale were averaged together to generate the total scores.

The Italian version of the Fear of Missing Out Scale (FoMOS) was used to assess disposition towards FoMO [23,35]. The scale comprises 10 items (e.g., “I fear others have more rewarding experiences than me”), rated on a five-point scale (from 1 = “not at all true of me” to 5 = “extremely true of me”). The reliability of the scale was good (α = 0.74).

The short Italian version of the 10-item Problematic Smartphone Use Scale [36,37] was used. Participants rate items on a six-point scale (from 1 = “strongly disagree” to 6 = “strongly agree”) with higher scores indicating higher PSU. A sample item is “I have used my smartphone longer than I had intended”. The scale reliability in the current sample was α = 0.82.

Participants’ daily social media use was assessed by asking them to indicate one of four options: “less than two hours”, “between two and four hours”, “between four and six hours”, and “more than six hours” [38].

### 2.3. Statistical Analyses

All the variables were screened for linearity, homoscedasticity, and homogeneity of variance. Additionally, univariate normality (skewness and kurtosis), multivariate outliers by computing Mahalanobis distance, and cases with missing values were checked. Following the general recommendations for univariate normality, the shape of the distributions of Machiavellianism and psychopathy were not severely non-normal since both the variables showed values of skewness and kurtosis >1.0 [39]. There were some missing data since answers to the items of the main variables were required to proceed in the survey.

Descriptive statistics and Pearson’s correlation analyses were computed. An independent-samples *t*-test was performed to compare the score variables between males and females. The internal reliability was obtained by computing Cronbach’s alpha (α). SPSS 26 package was used to run the preliminary statistical procedures. Structural equation modelling (SEM) was performed using Mplus 7.04 [40]. Hu and Bentler [41] recommended that multiple indices are used to evaluate the model fit (adopted cut-offs in brackets): the chi-squared (χ^2^) test value with the associated *p*-value (*p* > 0.05), comparative fit index (CFI ≥ 0.90), Tucker–Lewis Index (TLI ≥ 0.90), root-mean-square error of approximation (RMSEA ≤ 0.06), and its 90% confidence interval, and standardized root mean square residual (SRMR < 0.08).

The SEM model was estimated with the weighted least square mean and variance adjusted (WLSMV), treating the items as ordinal, involving a polychoric correlation matrix, and factor loadings using probit regression [42]. Finally, the partial mediating model and the full mediating model were compared to determine the mediating role of FoMO in the relationship between the DT of personality and PSU. The models were evaluated by using a chi-squared (χ^2^) difference test [39]. To establish significant differences between models, at least two out of three criteria had to be satisfied: Δχ^2^ significant at, *p* < 0.05, ΔCFI ≤ 0.005, and ΔRMSEA ≤ 0.010 [43]. All the analyses were controlled for age and gender [1], and daily social media use.

## 3. Results

The results of the correlational analysis, controlled by age, indicated that PSU was positively correlated with the main variables, excepted for psychopathy (see Table 1).

Next, an independent *t*-test was conducted to explore the presence of significant differences between males and females (see Table 2). The results indicated that females scored higher on PSU and daily social media use than males, while males reported higher scores on all the DT of personality traits, as well as on the FoMO.

The results shown in Table 3 summarize the mediation analysis as well as the direct and indirect effects of the tested model.

**Figure 1:**
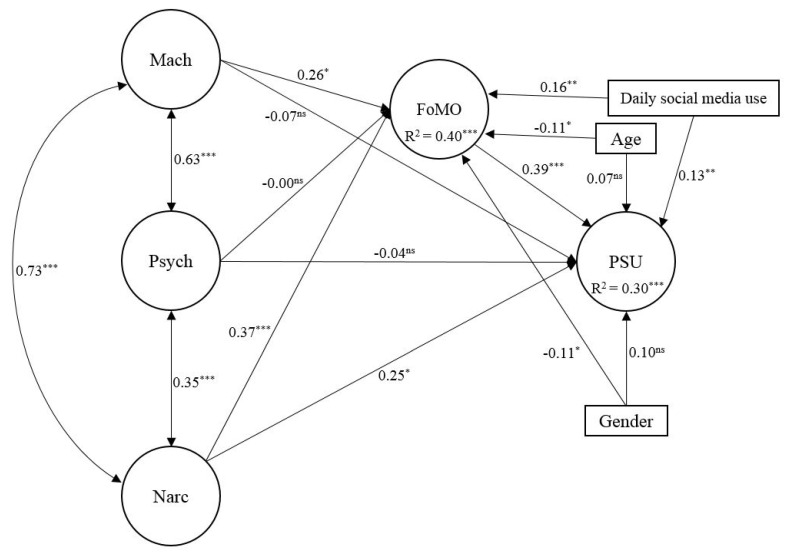
Results of the SEM analysis, including direct effects with respective standardized results and significance levels. *Note*. Mach = Machiavellianism; Psych = Psychopathy; Narc = Narcissism; FoMO = Fear of missing out; PSU = Problematic smartphone use. For clarity, item factor loadings, which were significant at *p* < 0.001, are omitted. * *p* < 0.05. ** *p* < 0.01. *** *p* < 0.001. ^ns^ = Non-significant.

The standardized results of the SEM analyses are presented in Figure 1. The research model provided a good fit to the data, robust χ^2^ (531, *N* = 455) = 1051.15, *p* < 0.001, CFI = 0.937, TLI = 0.929, RMSEA = 0.046, 90% CI [0.042, 0.051], SRMR = 0.079. FoMO and PSU accounted for 40% and 30% of the variance, respectively.

Narcissism had a direct effect on FoMO (*β* = 0.37, *SE* = 0.10, *t* = 3.68, *p* < 0.001) and PSU (*β* = 0.25, *SE* = 0.11, *t* = 2.30, *p* < 0.05), while Machiavellianism showed a weak association with FoMO (*β* = 0.26, *SE* = 0.14, *t* = 1.92, *p* = 0.05). FoMO had a significant effect on PSU (*β* = 0.39, *SE* = 0.07, *t* = 5.34, *p* < 0.001). Finally, FoMO partially mediated the relationship between narcissism and PSU (*β* = 0.14, *SE* = 0.05, *t* = 2.81, *p* < 0.01).

Finally, a fully mediated model and a partially mediated model were tested. Although the fully mediated model provided sufficient fit to the data (robust χ^2^ [534, *N* = 455] = 1048.84, *p* < 0.001, CFI = 0.937, TLI = 0.931, RMSEA = 0.046, 90% CI [0.042, 0.050], SRMR = 0.079), after comparing the two models, the results showed that the fully mediated model fitted the data worse (Δχ^2^(3) = 7.88, *p* = 0.049, ΔCFI = 0.000, ΔRMSEA = 0.000). Therefore, the partially mediating model was selected as final. After constraining the direct paths from DT of personality to PSU, the only significant associations were the relationship between narcissism and FoMO (*β* = 0.48, *SE* = 0.09, *t* = 5.50, *p* < 0.001), as well as between FoMO and PSU (*β* = 0.55, *SE* = 0.05, *t* = 11.30, *p* < 0.001).

## 4. Discussion

The present study investigated whether the association between DT personality traits and PSU was fully or partially explained by FoMO. In addition, the study attempted to verify the relationship between DT of personality (or single traits such as Machiavellianism and narcissism) and PSU. According to the findings, and consistent with the I-PACE model, personality traits, such as the DT of personality, were associated with PSU.

Partially in line with the hypotheses, the present results indicated a direct and significant association between narcissism and PSU. This finding is consistent with the existing literature, demonstrating that narcissistic individuals score higher on PSU [13]. Individuals with narcissistic traits may use smartphones for self-promotion and self-presentation in social media sites (such as Facebook, Instagram), given their disposition to these behaviors, and positive mood modification by satisfying the desired gratification may develop into PSU [32]. The results also demonstrated that narcissism was indirectly associated with PSU via FoMO. However, this association was not affected by gender and age.

The results indicated that higher narcissism, characterized by an unrealistically positive self-view, feelings of privilege, and a lack of regard for others, was associated with high FoMO. To the best of the authors’ knowledge, the present study is the first to investigate the role of the DT of personality as an antecedent of FoMO. It appears that narcissism is related to FoMO, which has been operationalized as the deficiencies of inherent needs for relatedness, competence, and autonomy [23]. Narcissists may use social media applications to fulfil their psychological needs originating from their antisocial personality such as the desire to be admired [30]. These findings add to those of prior studies indicating that narcissism appears to be an important trait that can lead to higher FoMO, which may turn into preoccupation with their social media profiles and others’ comments on their posts among social groups explored by using smartphone applications. This obsession may transform into PSU for a small number of individuals.

Moreover, Machiavellianism seems to be another antecedent of FoMO, although the findings of the present study showed a weak association. Machiavellianism involves manipulation and exploitation of others, and FoMO could promote such behavioral tendencies where potential benefits for an individual compensate potential damage or frustration to others [22] since FoMO has been also considered as a social needs fulfilment variable [24].

In the bivariate correlation, psychopathy was related to FoMO, but this finding was not consistently reflected in the SEM results. Additionally, psychopathy was not associated with PSU and this result contradicts other reported significant associations (e.g., [14,34]). One possible interpretation of this result could be that FoMO is often considered a product of depression symptoms [4,7]. Online environments may provide a medium for aggressive behavior that individuals who score higher in psychopathy are prone to engage in. Therefore, social media applications may facilitate psychopathic individuals to express their dark traits but not be affected by FoMO because they do not feel any apprehension concerning missing rewarding and pleasurable experiences to connect with others via social networks [23].

It should be noted that the present study is not without limitations. First, the cross-sectional design places restrictions on determining causal effects on the directionality of the relationships investigated. However, the results of the SEM analyses suggested some substantial and meaningful relationships between study variables. Second, the Dark Triad Dirty Dozen scale has been criticized for its brevity and lack of essential content. Additionally, since the present study used a self-report approach to collect data, Machiavellian people, due to this feature, may not have provided honest and sincere answers. Therefore, this DT trait may have been underestimated. Therefore, future studies should replicate the proposed research model using different (and more comprehensive) measures to assess DT personality traits. However, the results confirmed most of the hypotheses indicating that the Dark Triad Dirty Dozen scale appears to be an adequate instrument. Finally, another limitation is the age and gender of the participants. Since the mean age of our sample was 23.49 years and predominantly female, the sample mainly comprised female emerging adults; therefore, the findings cannot be generalized to the Italian population. Further research should be conducted with more representative samples to confirm the proposed relationships.

## 5. Conclusions

Overall, there are some important contributions offered by the present study. It partially replicated the results of a previous study, indicating that some DT of personality traits are associated with PSU [13]. More specifically, the study suggests that only narcissism, but not psychopathy, was directly associated with PSU. However, the present findings indicated, for the first time, that Machiavellianism and narcissism were antecedents of FoMO. However, further studies incorporating longitudinal designs should investigate these relationships by using different personality scales assessing DT traits. Finally, the present study was carried out using a non-clinical sample, therefore it would be interesting to test the same model with a clinical sample to learn more about risk factors associated with the development of disordered smartphone use.

## Figures and Tables

**Table 1:** Pearson’s correlations and descriptive statistics of the study variables among the full sample.

	1	2	3	4	5	6	7
1. Machiavellianism	-						
2. Psychopathy	0.44 ***	-					
3. Narcissism	0.57 ***	0.26 ***	-				
4. Fear of Missing Out	0.32 ***	0.16 **	0.42 ***	-			
5. Problematic Smartphone Use	0.17 ***	0.06	0.31 ***	0.36 ***	-		
6. Daily social media use (hours)	−0.01	−0.02	0.11 *	0.13 **	0.24 ***	-	
7. Age	−0.16	0.08	−0.00	−0.08	0.01	−0.18 ***	-
Skewness	1.50	1.09	0.98	0.88	0.53	−0.60	1.20
Kurtosis	2.84	1.11	0.60	0.75	−0.07	−1.03	1.95
*M*	1.41	1.76	1.93	1.94	2.63	3.15	23.49
*SD*	0.46	0.69	0.80	0.57	0.91	0.96	3.57

Note. * *p* < 0.05. ** *p* < 0.01. *** *p* < 0.001.

**Table 2:** Comparison of the total scores of the study variables between males and females.

	Males (*n* = 120)	Females (*n* = 335)	*t*-Test	Cohen’s *d*
1. Machiavellianism	1.59 (0.53)	1.35 (0.41)	5.03 ***	0.53
2. Psychopathy	1.98 (0.78)	1.68 (0.63)	4.15 ***	0.44
3. Narcissism	2.14 (0.84)	1.85 (0.78)	3.30 **	0.35
4. Fear of Missing Out	1.99 (0.56)	1.93 (0.58)	1.03	0.11
5. Problematic Smartphone Use	2.46 (0.85)	2.69 (0.93)	−2.34 *	−0.25
6. Daily social media use (hours)	2.73 (1.02)	3.30 (0.90)	−5.71 ***	0.59

Note. *M* (*SD*). * *p* < 0.05. ** *p* < 0.01. *** *p* < 0.001.

**Table 3:** Mediation analysis, total, indirect, and direct effects with standardized estimates among the whole sample.

Pathway	Estimate	*SE*	Z	*p*
Machiavellianism				
🡺 PSU (total effect)	0.03	0.14	0.25	0.800
🡺 PSU (indirect effect)	0.10	0.05	1.85	0.064
🡺 PSU (direct effect)	−0.07	0.15	−0.44	0.659
Psychopathy				
🡺 PSU (total effect)	−0.04	0.08	−0.55	0.584
🡺 PSU (indirect effect)	−0.00	0.03	−0.06	0.955
🡺 PSU (direct effect)	−0.04	0.08	−0.51	0.607
Narcissism				
🡺 PSU (total effect)	0.39	0.09	4.14	0.000
🡺 PSU (indirect effect)	0.14	0.05	2.81	0.005
🡺 PSU (direct effect)	0.25	0.11	2.30	0.021

Note. PSU = Problematic smartphone use.

## Data Availability

Data are available on request due to ethical issues. The data presented in this study are available on request from the corresponding author.
